# Supplementary material for: In Situ Electrochemical Synthesis of Squamous-like Cu2S Induced by Sulfate-Reducing Bacteria as a Fenton-like Catalyst in Wastewater Treatment: Catalytic Performance and Mechanism
Source: Nanomaterials (Basel). 2024 Apr 2;14(7):621. doi: 10.3390/nano14070621 (PMC11013312; doi:10.3390/nano14070621)
Supplement: Supplementary file 1 [file nanomaterials-14-00621-s001.zip › nanomaterials-2916491-supplementary.pdf]

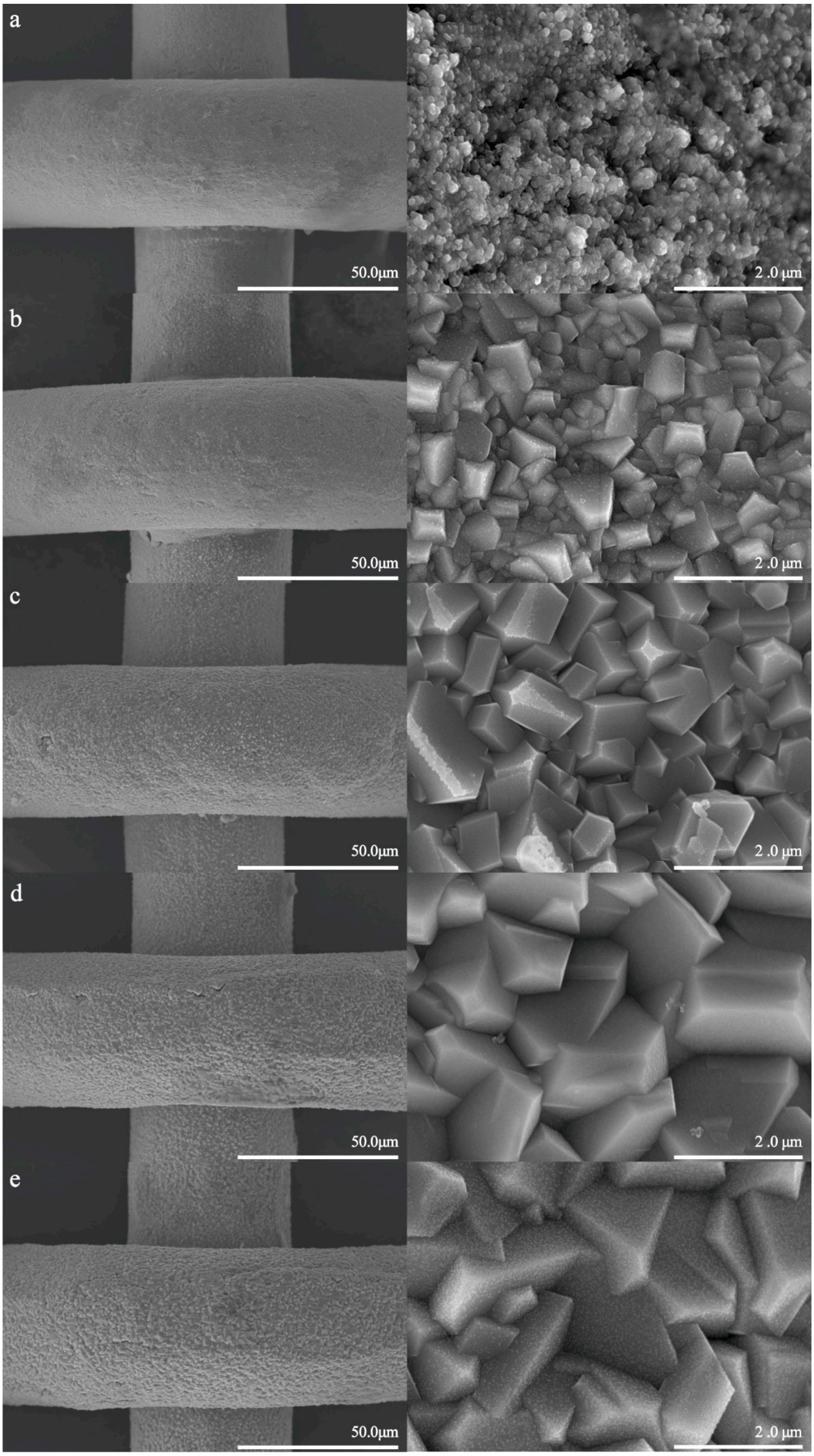

Figure S1. SEM of CSCM-SRB with same electrolytic time (900 s) and different current density: 0.25 mA/cm^2 (a); 1.25 mA/cm^2 (b); 2.5 mA/cm^2 (c); 3.75 mA/cm^2 (d); 5 mA/cm^2 (e)

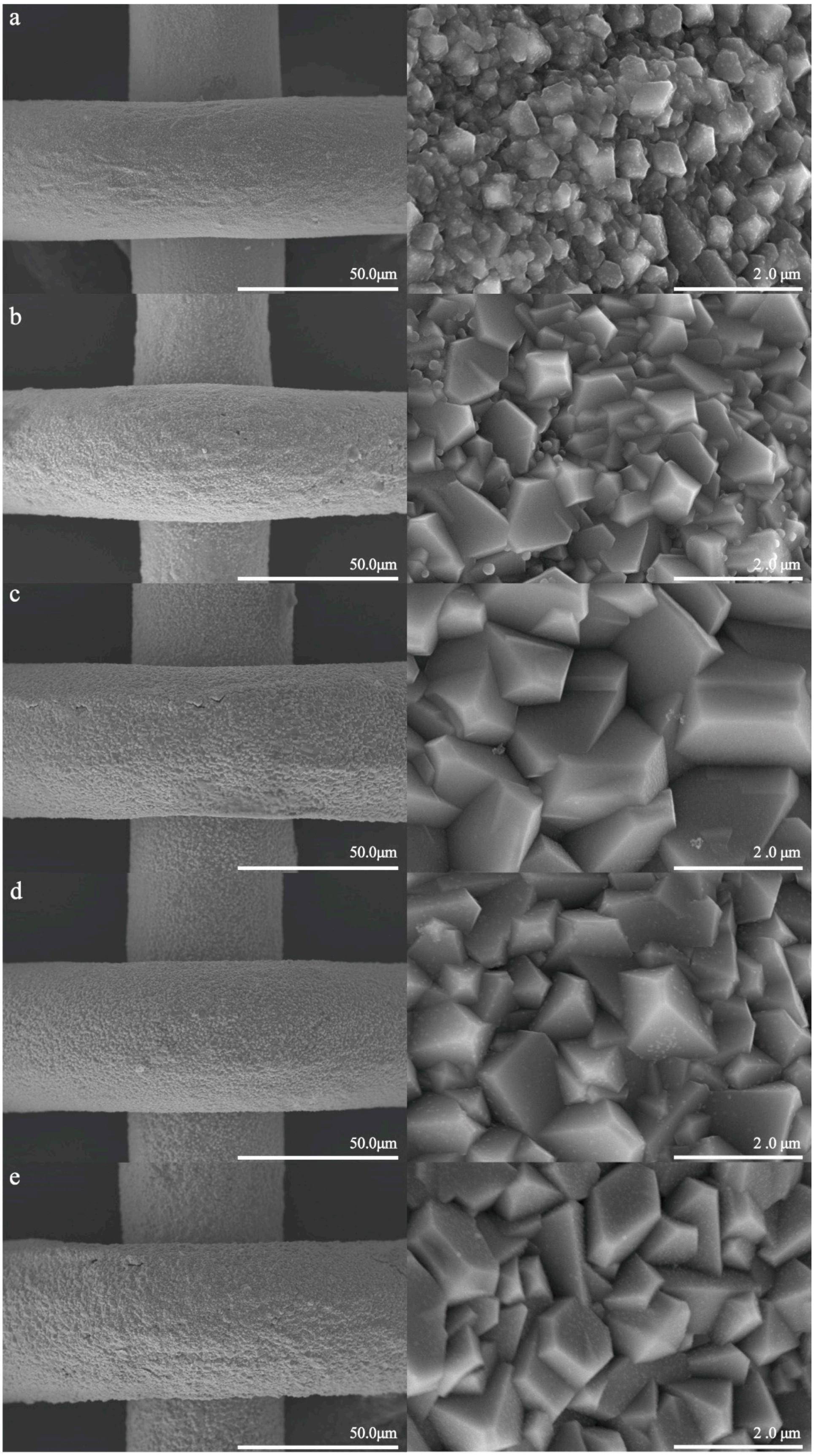

Figure S2. SEM of CSCM-SRB with same current density (3.75 mA/cm^2) and different electrolytic time: 300 s (a); 600 s (b); 900 s (c); 1200 s (d); 1500 s (e)
